# Translating genome-wide association studies at multiple scales: Drug target prioritization, cellular architectures, and organ imaging

**DOI:** 10.1016/j.xgen.2026.101282

**Published:** 2026-06-17

**Authors:** Benedetta Felici, Siyuan Chen, Meng Yuan, Xilin Jiang, Samantha Ip, James H.F. Rudd, Michael Inouye

**Affiliations:** 1 Cambridge Baker Systems Genomics Initiative, Department of Public Health and Primary Care, University of Cambridge, Cambridge, UK; 2 British Heart Foundation Cardiovascular Epidemiology Unit, Department of Public Health and Primary Care, University of Cambridge, Cambridge, UK; 3 Victor Phillip Dahdaleh Heart and Lung Research Institute, University of Cambridge, Cambridge, UK; 4 Department of Epidemiology, Harvard T.H. Chan School of Public Health, Boston, MA, USA; 5 British Heart Foundation Centre of Research Excellence, University of Cambridge, Cambridge, UK; 6 Cambridge Centre for AI in Medicine, Department of Applied Mathematics and Theoretical Physics, University of Cambridge, Cambridge, UK; 7 Centre for Cancer Genetic Epidemiology, Department of Public Health and Primary Care, University of Cambridge, Cambridge, UK; 8 Health Data Research UK Cambridge, Wellcome Genome Campus and University of Cambridge, Cambridge, UK; 9 Cambridge Baker Systems Genomics Initiative, Baker Heart & Diabetes Institute, Melbourne, VIC, Australia

## Abstract

With a vast corpus of findings from nearly two decades of genome-wide association studies (GWASs), many studies now focus on translating these genetic associations into biological insights at multiple scales, from proteins and cells to entire organs. This approach will help build the foundation for the next generation of treatments. In this review, we highlight key recent studies that have informed target prioritization and drug repurposing, linked genetic variants to gene regulation in cellular contexts, and uncovered the genetic architecture of organ structure and function. Nearly 25 years after the initial draft of the human genome, it is clear that genomics is driving tangible advances in therapies and opening new ways to understand multi-scale biology.

## Graphical abstract

**Figure:**
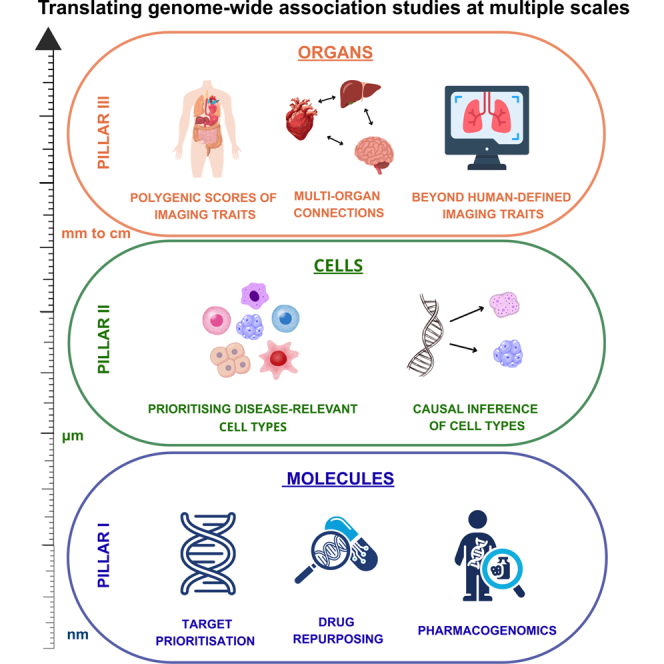

## Teaser

This review covers how the results of genome-wide association studies are now utilized at multiple scales, informing our etiological understanding at the levels of molecules, cells, and organs. It covers computational and conceptual advances that build on multi-omics technologies and may lead to the development of a new generation of therapies to improve human health.

## Introduction

In the past decade, research into the human genomics of common diseases has shifted from locus discovery to functional interpretation, with various advances including quantitative trait locus (QTL) mapping,^1^ fine-mapping^2^^,^^3^ and multi-ancestry studies. These developments have enabled researchers to begin bridging the gap between statistical associations and biological mechanisms, paving the way for applications in drug discovery, risk prediction, and precision medicine.

Signals from human genetic studies are being harnessed for therapeutic target prioritization by intersecting with druggable gene databases, while in imaging genetics, the effects of genetic variants on organ structure and function are elucidating early disease manifestations.^4^^,^^5^^,^^6^^,^^7^^,^^8^ At the cellular level, single-cell RNA sequencing and spatial transcriptomics now enable the mapping of genome-wide association study (GWAS) loci to specific cell types, refining our understanding of disease-relevant cellular architectures.^9^ Beyond genetic discovery, GWAS findings are increasingly leveraged for translational applications. Polygenic scores (PGSs) have entered clinical evaluation,^10^ and provisional guidance has been issued for their use in clinical risk prediction; e.g., for cardiovascular disease.^11^^,^^12^

While the steps in the translational pathway for any new therapy are highly specific (and largely non-linear), a typical path to the clinic for GWAS findings can be summarized as beginning with the prioritization of (a) causal gene(s) at a locus. The following steps generally include characterizing the specific conditions of the gene’s relevant effect (e.g., cell type, tissue, life stage); performing *in vitro* and *in vivo* mechanistic studies to validate the gene’s causal effects on molecular pathways; screening, design, and optimization for candidate drugs that modulate the target effectively and safely; preclinical studies to select the best candidate; and finally clinical trials to test safety, dosage and efficacy in phases I, II, and III. Therapies that fulfill this pathway are then considered for regulatory approval and, if successful, enter a period of post-market surveillance.

**Figure 1:**
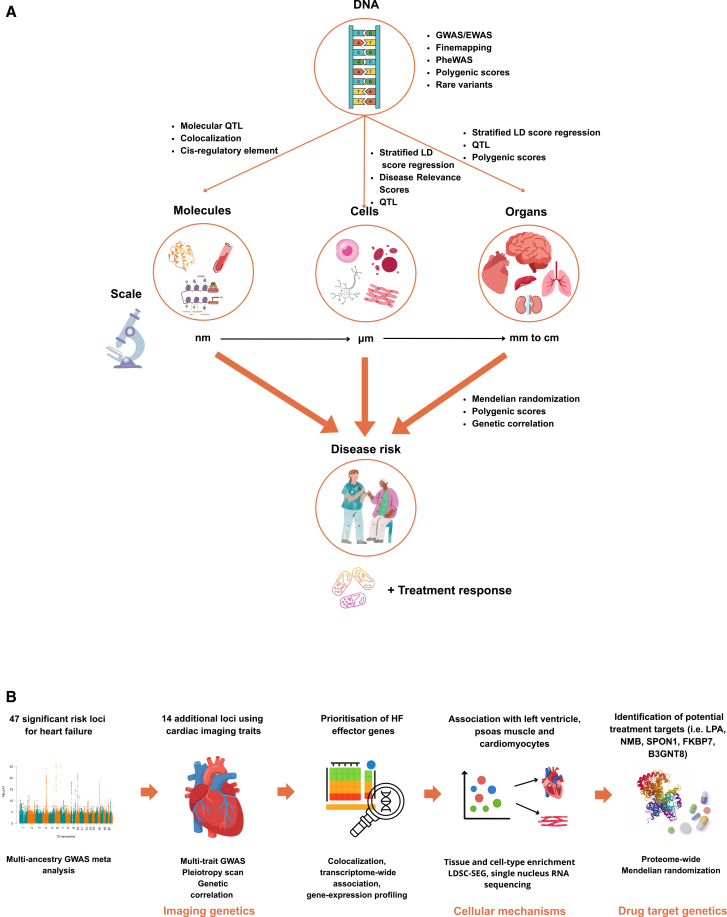
Integration of multi-scale data to uncover drug targets and disease etiology (A) Schematic of multi-scale, multi-modal approaches. (B) A case study based on Levin et al.,^13^ demonstrating how integrated analysis of cardiac imaging, cellular, and tissue data enabled the identification of potential therapeutic targets for heart failure.

This review focuses on three key post-GWAS research areas that can have an impact throughout the translational pathway: (1) therapeutic target prioritization, where genetic associations guide drug discovery; (2) cellular architectures of disease, leveraging single-cell technologies to dissect the cell type-specific mechanisms of GWAS signals; and (3) imaging genetics, which connects genetic variants to organ-level physiology and function (Figure 1A). By synthesizing recent advances within and between these areas, we highlight how post-GWAS studies are driving the translation of genomics into new tools and therapies that could ultimately improve patient outcomes and population health.

## Pillar I: Genetics for therapeutic target prioritization and drug discovery

Selecting a therapeutic target with the highest likelihood of success is crucial; human genetic evidence, particularly from GWASs, is a key tool in this process. Indeed, successfully bringing a drug through all stages of development is extremely challenging, typically taking 10–15 years and costing more than US$2 billion.^14^ Despite rising costs, drugs frequently fail, principally due to lack of efficacy (∼50% of drug failures) but also lack of safety (∼25% of drug failures). Additionally, failure commonly occurs during the later stages of development (i.e., phase II and III clinical trials), which is problematic.^15^^,^^16^

Multiple studies have demonstrated that drug targets supported by human genetic evidence are more likely to succeed in all stages of drug development.^17^^,^^18^ GWASs themselves have been shown to prioritize existing drug targets. For example, glitazone and sulfonylurea, used to treat type 2 diabetes, were validated *a posteriori* through GWASs.^4^ The same is true for the *HMGCR* locus and cholesterol, now widely treated with statins, formally known as HMG-CoA reductase inhibitors.^19^

In the decade prior to 2022, the proportion of approved drugs with human genetic support was ∼60%, with some annual fluctuation (range approximately 41% and 72%).^20^ There has also recently been a trend of increasing evidence of human genetic support prior to drug approval (perhaps the most relevant metric), from 53% (2013–2017) to 63% (2018–2022).^20^ This is despite a prolonged time gap between genetic target identification and eventual drug approval, with a recently reported median of 25 years according to Trajanoska et al.^21^ Consistent with this, Minikel et al.^22^ reported that active programs showed only marginally more genetic support than historical programs and less than for launched drugs, with only a small part of druggable genetically supported gene-indication pairs having been carried forward.^22^ Importantly, Minikel et al. also demonstrated that therapeutic targets supported by genetic information were 2.6 times more likely to succeed than those without such evidence.^22^

Razuvayevskaya et al. further demonstrated that poor genetic support is associated with clinical trial failures due to poor therapeutic efficacy, while drug targets that are highly constrained (i.e., intolerant of mutational change) in humans frequently underlie clinical trial failures due to safety concerns.^23^ Given the potential benefit of systematically incorporating genetic data into therapeutic development, Namba et al. proposed guidelines for genomics-driven drug discovery in a cross-population meta-analysis, focusing on overlap enrichment analysis, endophenotype Mendelian randomization, and negative correlation tests.^24^

Rare variants have been particularly useful in drug development. It has been reported that target genes for approved drugs are more enriched in rare variants compared to common variants,^25^ and, similarly, the genetic variants most strongly linked to drug approvals were associated with severe genetic disorders and altered amino acids,^26^ where approximately 95.7% of functionally important variants are rare.^27^

*PCSK9* and *ANGPTL3* are two examples of how rare genetic mutations can guide drug discovery. During the early 2000s, a study on a French family affected by familial hypercholesterolemia led to the discovery of a gain-of-function mutation in *PCSK9,*^25^ while African Americans with extremely low cholesterol levels and cardiovascular disease risk were found to carry a loss-of-function mutation in the same gene.^28^ Today, the PCSK9 inhibitors alirocumab (Praluent) and evolocumab (Repatha) are US Food and Drug Administration approved medications and options for patients who cannot take statins. At the *ANGPTL3* locus, large whole-exome sequencing discovered a loss-of-function variant associated with significantly lower cholesterol levels as well as reduced coronary artery disease risk.^29^ In 2021, the US Food and Drug Administration (FDA) approved an ANGPTL3 inhibitor (a monoclonal antibody, evinacumab) to reduce high-density lipoprotein (HDL), low-density lipoprotein (LDL), triglyceride levels, and cardiovascular disease risk.^30^

Disease risk is often shaped by both rare and common genetic variants, with the latter distributed broadly across the genome. According to the omnigenic model, genes can influence traits either directly or indirectly.^31^ Core genes act through more explicit disease-relevant biological pathways, while peripheral genes implicitly affect disease risk via regulatory mechanisms or interactions with core genes.^31^^,^^32^ This framework appears to be particularly relevant for traits that lie between complex and Mendelian genetics, including blood cell phenotypes.^33^ The distinction between peripheral and core genes is especially important for target prioritization. A GWAS in combination with post-GWAS analysis, such as protein-protein interaction networks or more general network propagation methods, have been effective in identifying core genes, possibly more enriched for drug targets.^32^^,^^34^ More in general, using integrative tools such as the priority index^35^ and the polygenic priority score,^36^ which combines a GWAS with functional annotations, expression data, and network evidence, have demonstrated to be important in gene and target prioritization.

An exemplar of GWAS-informed drug development is the association identified between the TYK2 locus and psoriasis.^37^ A GWAS informed the prioritization of TYK2 as a drug target for multiple autoimmune indications and, over a decade after the original GWAS, the FDA approved an oral TYK2 inhibitor for psoriasis in 2022. A GWAS also uncovered *BCL11A* as a suppressor of fetal hemoglobin and a potential therapeutic target in sickle cell anemia,^38^ which eventually led to the world’s first medicine based on gene editing (Casgevy).

Beyond drug discovery and target validation, a GWAS has other therapeutic applications. It has deepened our understanding of disease mechanisms and has driven the development of new genetic tools, such as phenome-wide association study (PheWAS), Mendelian randomization (MR), and PGSs, which have useful therapeutic applications, from drug repurposing to pharmagenomics.^4^^,^^39^^,^^40^^,^^41^

## Drug repurposing

Drug repurposing uses drugs developed for one disease to treat another. The phenomenon of pleiotropy, particularly region- and gene-level pleiotropy, can inform drug repositioning. Several studies have used GWASs to retrospectively investigate the extent to which existing drug indications have consistent or inconsistent GWAS associations, with the latter potentially creating opportunities for repurposing.^4^^,^^19^ Using MR, Finan et al. identified 144 approved therapeutic indications with repurposing potential due to divergence from GWAS findings.^4^

GWASs have driven the repurposing of multiple drugs. Interleukin-17A (IL-17A) inhibitors were initially developed for psoriasis, rheumatoid arthritis, and uveitis and were later extended to ankylosing spondylitis based on a GWAS signal downstream of *IL23R*,^42^ a key regulator of IL-17-producing Th17 cells*.* Similarly, as part of the IL-17/IL-23 immune axis, IL-23 inhibition for psoriasis was repurposed for Crohn’s disease based on one of the very first GWAS signals.^18^^,^^43^ MR has been an informative extension of GWAS when used for drug repurposing. For instance, Yin et al. used the *CASR* variant rs1801725 to show that genetically increased serum calcium levels lead to higher odds of migraine, supporting the repurposing of the CaSR antagonist (cinacalcet), developed for treatment of hyperparathyroidism, against migraine treatment.^44^ Similarly, many GWASs and MR studies have implicated IL-6 signaling in atherosclerotic disease, providing key evidence in support of repositioning IL-6 inhibitors (e.g., tocilizumab and sarilumab), currently used for various autoimmune inflammatory diseases, to cardiovascular diseases.^45^ Published trials appear to support this repurposing.^46^^,^^47^

## Pharmacogenomics

Genetic information also helps explain variability in drug response between individuals, a field known as pharmacogenomics. In the UK, 58% of patients are prescribed at least one drug with a known pharmacogenetic variant, increasing to nearly 90% for patients over 70 years of age.^48^ Pharmacogenomics encompasses various areas of research, including determining appropriate drug dosages, preventing adverse reactions, and improving treatment efficacy. For these purposes, many resources are available, such as PharmVar,^49^ Pharmacogenomics Knowledge Base (PharmGKB),^50^ and the FDA’s table of pharmacogenomic associations.^51^

Indeed, the optimal drug dosage may be improved using genetic variants; for example, dosing of the anticoagulant warfarin, which is influenced by variants in both *CYP2C9* and *VKORC1*.^52^ Among anticancer drugs, dosing of thiopurines is impacted by genetic alterations in *TPMT* and *NUDT15*, fluoropyrimidines by *DPYD* variants, and irinotecan by *UGT1A1* polymorphisms.^52^ Tacrolimus, used to prevent rejection in organ transplant recipients, is affected by a genetic variant in *CYP3A5*, which accounts for 39% of the differences in required dosing.^53^ Birdwell et al. also considered ancestry differences and noted that the higher frequency of *CYP3A5∗1* in individuals of African ancestries had a higher required daily dose.^53^

Some MR studies have directly influenced the design of clinical trials. The Lp(a) HORIZON phase III trial enrolled individuals with relatively high Lp(a), following the results of MR studies.^40^^,^^54^^,^^55^ MR found that, in primary prevention, achieving at least a 20% relative reduction in cardiovascular events required an Lp(a) reduction of 65–80 mg/dL.^40^^,^^54^^,^^55^ In secondary prevention, however, a reduction of 50 mg/dL in Lp(a) might be sufficient to achieve similar results.^54^

GWASs have informed drug safety and adverse drug reactions. For example, SNPs in *SLCO1B1* are linked to a higher risk of statin-induced myopathy.^56^ A particular type of adverse drug reaction is drug hypersensitivity reactions (DHRs), often influenced by genetic variants in the human leukocyte antigen (HLA) region. For example, hypersensitivity to abacavir is associated with HLAB∗57:01 and hypersensitivity to carbamazepine with HLA-B∗15:02 and HLA-A∗31:01^57^; while DHRs to allopurinol are linked to HLA-B∗58:01 and ADRs for flucloxacillin to HLA-B∗57:01.^57^ In the case of nevirapine DHRs, implicated variants include HLA-B∗35:05, HLA-C∗04:01, and HLA-DRB1∗01:01.^57^ Finally, DHRs to amoxicillin-clavulanate are connected with HLAA∗02:01, HLA-DRB1∗15:01, and HLA-DQB1∗06:02 and for dapsone and lapatinib with HLA-B∗13:01 and HLA-DQA1∗02:01, respectively.^57^ Moreover, carriers of HLA-B∗5701 have a much higher risk of drug-induced liver injury with flucloxacillin.^58^ HLA-DQA1∗05 has been linked to the development of antibodies against anti-tumor necrosis factor therapies, which are used to treat immune-mediated diseases such as Crohn’s disease.^59^ GWASs and MR may also inform risk minimization of unintended side effects, such as sclerostin inhibitors approved for osteoporosis, which were found to increase cardiovascular disease risk via SNPs in *SOST*.^60^

In informing drug efficacy, the action of olaparib (a targeted cancer drug) was found to be influenced by *BRCA1* and *BRCA2* mutations, and therapeutic response to clopidogrel (an antiplatelet agent) is affected by *CYP2C19∗2* or *CYP2C19∗3* variants.^52^ In addition, variants in *CYP2D6*, which show markedly different frequencies across ancestries (ranging from 0% in West Africa to 12% in the UK), affect the response to codeine, potentially leading to toxicity in ultra-rapid metabolizers.^52^ Nearly half of heart failure with reduced ejection fraction (HFrEF) patients on the β-blocker bucindolol who were β_1_389 Arg homozygotes experienced a significant reduction in their chance of developing atrial fibrillation.^61^

In this context, PGSs may also have utility. Several trials, such as the ODYSSEY OUTCOMES trial and the FOURIER trial, have shown that individuals with a high PGS for coronary artery disease derive the greatest benefits from PCSK9 inhibitors.^39^^,^^62^ Similar results were found for statins, with individuals in the top quintile of a PGS for coronary disease having the strongest relative risk reduction of heart attack and cardiovascular death with statin therapy.^63^ The use of PGSs to guide clinical trial design and improve the efficacy of new medicines, through enhanced prognostic and predictive enrichment, may hold substantial promise.^64^

To date, most pharmacogenomics research has focused on disease susceptibility. However, given the limited genetic overlap between susceptibility and disease-specific mortality,^65^ there is a clear need to expand research toward understanding how genetic predictors of disease survival and progression, beyond disease susceptibility, interact with treatment response.

## Pillar II: Cellular architectures of disease

The human genetic-guided therapeutic target prioritization, described above, depends crucially on the biological context of a target’s effect. Single-cell atlases and population-scale single-cell RNA sequencing (scRNA-seq) datasets have rapidly expanded over the last decade, including Human Cell Atlas,^66^ OneK1K,^67^ and the ROSMAP project.^68^^,^^69^ Single-cell atlases of chromatin accessibility for *cis*-regulatory elements have been constructed in parallel.^70^^,^^71^ These studies enhance our ability to dissect the cellular drivers of disease. Data from these cellular studies can be integrated with genetic information to bridge the gap between GWASs and mechanistic biology. GWAS-identified alleles are frequently located in non-coding regions, which influence phenotypic variation through multiple gene-regulatory elements, whose activity may differ across tissues and cell types.^70^^,^^72^^,^^73^ By integrating single-cell datasets and germline genetic information, researchers can now pinpoint cell types that mediate the genetic basis of complex traits and identify candidate effector genes as potential drug targets in a cell type-specific context. For example, by identifying cell types in the central nervous system rather than adipose tissue, obesity is shown to be a genetically neurological/behavioral disorder,^74^^,^^75^ validating the mechanism of GLP-1 agonists (like Wegovy/Ozempic), which act on the brain to control satiety. GWASs showed that microglial receptors were strongly associated with increased Alzheimer’s disease risk,^76^^,^^77^^,^^78^ leading to further investigations that revealed a unique microglia type associated with restricting the development of Alzheimer’s disease^79^ and potential targeted therapy.^80^ With methodologies rapidly changing, we summarize strategies for linking GWAS signals to cell type and cellular characteristics as well as their potential use cases.

## Prioritizing disease-relevant cell types via heritability enrichment

A commonly used approach to link GWAS variants to cell types is stratified linkage disequilibrium score regression (S-LDSC), a framework initially developed by Finucane et al.^75^ Rather than mapping individual variants to specific cells, these methods identify cell types by evaluating the enrichment of trait heritability within genomic regions characterized by cell type-specific expression or chromatin accessibility.^81^ Building on this foundation, subsequent methodological advances have significantly refined precision and applicability.

Under this framework, publicly available single-cell data can be used to create cell type annotations for SNPs, which can then be linked to complex traits by evaluating how GWAS signals are enriched across these cell type annotations. For example, CELLECT^82^ enhances cell type prioritization by leveraging multiple expression specificity metrics derived from scRNA-seq data, including differential expression T-statistic, gene enrichment score, expression proportion, and normalized specificity. CELLECT supports multiple genetic prioritization models, including S-LDSC^75^ and MAGMA^83^ covariate analysis, for inference of disease-relevant cell types. Jagadeesh et al. introduced sc-linker, which uses continuous cell type annotations and tissue-specific enhancer-gene linking strategies to model the activity of regulatory elements across cell states, thereby boosting statistical power.^78^ Additionally, Kim et al.^84^ combined S-LDSC with single-cell assay for transposase-accessible chromatin using sequencing (scATAC-seq) to map GWAS signals to cell type-specific open chromatin regions. Recently, spatial transcriptomic annotations^85^ have expanded the framework to reveal spatial gradients of genetic heritability in organs such as the brain and liver, highlighting the importance of contextualizing cell type-specific signals within their native tissue environments. Additionally, methods like scDRS use GWAS data to identify cell types that exhibit excess expression across disease-associated genes.^86^

These methods have had wide-ranging applications and enabled research that bridges cell types and complex traits. A study integrating a hypothalamus spatio-cellular map and body mass index (BMI) GWAS showed that the energy balance in humans is mostly neuron centric and regionally organized, with a strong enrichment in mid-hypothalamic neuronal populations.^87^ In that study, both well-established causal genes of obesity (*MC4R*, *PCSK1*, *POMC*, and *CALCR*) and novel genes (*BSN* and *CORO1A*) were identified as effector genes driving cell type associations. Etiological heterogeneity in type 2 diabetes (T2D) was found through differential enrichment of cell type-specific open chromatin regions, including pancreatic islets, adipocytes, endothelial cells, and enteroendocrine cells, across nonoverlapping clusters of T2D GWAS signals.^70^^,^^88^ Similarly, the genetic risk of coronary artery disease was found to be predominantly mediated through smooth muscle and endothelial cells, with a more prominent role observed for genes that participate in pathological changes compared to those that maintain a healthy cell identity.^89^ In osteoarthritis, prioritizing cell types with GWAS findings showed mechanistic differences.^90^ For example, while chondrogenesis cell types were involved in all sites of osteoarthritis, interzone chondrocytes specifically contributed to risk of total hip replacement, and osteogenesis cell types were involved in risk of total hip replacement, hip osteoarthritis, and finger osteoarthritis but not in knee osteoarthritis and total knee replacement. Using GWAS data and scRNA-seq or scATAC-seq data, atlases of cellular process-disease associations^70^^,^^91^ have been created. These integrative genetic and single-cell analyses highlight how mapping disease-associated variants onto precise cellular contexts can reveal cell type-specific mechanisms and may potentially inform targeted therapeutic strategies.

However, the heritability enrichment-based method requires high polygenicity to yield stable enrichment estimates. Furthermore, the statistical power of these methods is sensitive to annotation size, with methods primarily used under annotation sizes >0.5% SNPs. If the genomic footprint of the linked enhancers or cell type-specific peaks is too small, then heritability estimates can become unstable, resulting in inflated type 1 error.^92^ While these integrative frameworks are optimized for steady cell types, Rumker et al. developed genotype-neighborhood associations, allowing exploration beyond cell types by identifying genetic variants influencing the abundance of cell states.^93^

## Single-cell QTLs and causal inference of cell types

The emergence of population-level scRNA-seq datasets enables the identification of cell type-specific expression QTLs (sc-eQTLs), offering unprecedented resolution to link genetic variants to gene regulation in diverse cellular contexts. However, sc-eQTL mapping faces unique statistical challenges due to the sparsity of single-cell data. Pseudobulk approaches aggregate counts across cells of the same cell type per individual to stabilize the variance, while cell-level models, such as Poisson mixed effects, can better capture cell-to-cell heterogeneity or continuous cell states but require higher computational overhead and careful handling of zero values.^94^ Landmark studies, such as sc-eQTL mapping in over one million peripheral blood mononuclear cells^67^ and cell-type-specific cis-eQTLs mapping in eight human brain cell types,^95^ have demonstrated that genetic control of gene expression and transcriptional regulation is highly dynamic and heterogeneous across cell types. In the brain’s neocortex, Fujita et al. identified ∼10,000 and ∼8,000 genes targeted by *cis*-eQTLs at the cell type and cell subtype level, respectively.^96^ Through colocalization of genetic risk variants and single-cell *cis*-eQTL, these studies also showed cell type-specific mechanisms for autoimmune diseases and identified novel risk genes for psychiatric and neurological disorders.

While large-scale scRNA-seq data are still quite limited, decomposition of bulk RNA-seq datasets can be a useful approach; for example, in cell-type-interaction QTL mapping.^97^ Methods such as IBSEP^98^ and JOBS^99^ integrate both scRNA-seq and bulk RNA-seq data to enhance sc-eQTL prioritization. Evaluating whether tissue-level eQTLs are more likely to overlap with cell type-specific *cis*-regulatory elements, identified by external scATAC datasets, can also help to prioritize cell types associated with GWAS traits.^100^^,^^101^^,^^102^^,^^103^ Furthermore, the direct mapping of single-cell chromatin accessibility QTLs from scATAC-seq provides a high-resolution view of genetic effects on chromatin architecture.^104^ Most current research focuses on local *cis*-eQTLs, but there is increasing interest in distant *trans*-eQTLs. Although harder to detect due to the heavy multiple-testing burden, *trans*-effects are often more cell type specific and provide a broader view of regulatory networks. Large-scale consortia, including sc-eQTLGen and MetaBrain, worked on mapping these distal *trans*-sc-eQTLs at scale.^105^^,^^106^ 737 *trans*-eQTLs were identified by de Klein et al. in 7 central nervous system regions.^107^

Downstream of QTL discovery, post-QTL methods, such as fine-mapping and colocalization, are essential for identifying true causal variants. Fine-mapping prioritizes the most likely functional variants within a locus, while colocalization assesses whether the same genetic signal underlies both a QTL and a GWAS trait, reducing the risk of coincidental overlaps. Through colocalization of single-cell eQTL and GWAS loci, Yazar et al. found that 19% of *cis*-eQTLs share the same causal locus as a GWAS risk association.^67^ Additionally, single-cell QTLs provide an important resource for causal inference for post-GWAS functional genomics. By combining sc-eQTLs with MR and colocalization, researchers can prioritize effector genes and cell types underlying GWAS signals.^108^^,^^109^ Recent studies have leveraged this approach to uncover cell type-specific mechanisms across diverse traits, such as a studies by Ying et al., which identified 132 putative causal genes across 14 immune cell types associated with COVID-19 outcomes,^110^ and a study by Wu et al., which identified 162 and 80 genes associated with T2D and coronary artery disease, respectively, with many exhibiting CD4^+^ T cell specificity.^111^ Similarly, Hao et al.^112^ prioritized distinct brain cell types mediating the causal effects of BMI on 18 diseases, and Wang et al.^99^ revealed novel rheumatoid arthritis risk genes, such as the inflammasome-associated *DDX17*, across immune cell types. Large-scale colocalization analyses further identified 501 gene-cell type pairs linked to 30 central nervous system phenotypes, including MR evidence that *EGFR* expression increases Alzheimer’s disease risk, with potential repurposing of EGFR inhibitors.^113^ These studies illustrate the power of sc-eQTLs in dissecting the cellular context of complex trait architecture, bridging genetic associations to potentially translational insights. While sc-eQTLs and MR provide strong evidence for effector genes, caution should be exercised due to pleiotropic effects, where a single variant influences multiple genes or cell types simultaneously, potentially confounding causal interpretations.

## Pillar III: Imaging connects genetic variants to organ-level physiology and function traits

Recently, the UK Biobank, the world’s largest imaging project, achieved its milestone of gathering brain, body, and bone scans from 100,000 volunteers.^114^ The development of large imaging datasets with matched genetics, such as the UK Biobank, have enabled researchers to explore the genetic basis of organ structures and link these endophenotypes to disease development (Table S1). Imaging-derived phenotypes (IDPs) are extracted from medical images, which are rich in information about organ function and structure, making them valuable tools for understanding disease etiology, making diagnoses, and enhancing risk prediction. As intermediate phenotypes, they also allow us to clarify the physiological implications and potentially causal mechanisms on the path between GWAS signals and disease events. In addition, they have proven useful to uncover disease subtypes, identifying distinct pathological mechanisms and genetic architectures.^115^ Beyond this, studying the genetics of endophenotypes can offer greater statistical power, both because genetic effects tend to be stronger for traits that are “closer” to gene action^115^^,^^116^ and because imaging phenotypes are continuous traits that can be measured in the general population and not necessarily in disease-ascertained cohorts. Moreover, disease cases are often heterogeneous, comprising multiple subtypes with both shared and distinct genetic risk factors. Continuous imaging phenotypes can more precisely delineate this heterogeneity by representing disease variation along a spectrum rather than as a binary case-control distinction or a small set of discrete subtypes.

Initially, GWAS of imaging traits focused mainly on the brain. For brain structure, many loci have been identified, including those for intracranial volume,^117^ cortical structure,^118^ white matter microstructure,^119^ and brain growth and atrophy.^120^ More recently, new imaging modalities, such as quantitative susceptibility mapping (QSM) using MRI and amyloid positron emission tomography (PET) imaging, have enabled the identification of genetic variants associated with specific brain constituents, such as iron, calcium, myelin, and β-amyloid beta (Aβ) accumulation.^121^^,^^122^ For the latter, ancestry-specific effects were observed for the APOE ϵ4 and ε2 isoforms.^122^

Brain structural traits have been shown to be genetically correlated with numerous phenotypes, thereby potentially serving as endophenotypes, such as intracranial volume and Alzheimer’s or Parkinson’s disease.^117^ Cortical structures, such as surface area, have genetic correlations with general cognitive function, Parkinson’s disease, insomnia, attention-deficit/hyperactivity disorder (ADHD), depression, and neurotic traits.^118^ White matter microstructure is genetically correlated with stroke, ADHD, schizophrenia, and major depressive disorder,^119^ while brain growth and atrophy are correlated with various psychiatric and neurodegenerative features as well as with height, BMI, and smoking.^120^

Also, numerous GWASs have been conducted on cardiac structures. One hundred and thirty independent genetic loci have been identified for right heart measurements, many proximal to genes implicated in congenital heart disease,^8^ while 72 loci have been found for left ventricular regional wall thickness traits and related to heart development and contraction pathways.^123^ GWASs identified 11 and 21 loci associated with descending aorta distensibility and strain and 12 and 26 loci related to the same structures in the ascending aorta, some of which are proximal to genes for elastogenesis and atherosclerosis.^124^ Levin et al., through a GWAS of heart failure, further demonstrated that adding cardiac imaging traits as part of a multivariate GWAS increased the number of discovered loci from 47 to 61.^13^ Three-dimensional imaging has also been shown to be a powerful approach to uncovering genetic associations, as it captures more heritability than traditional 2D imaging methods.^125^ A similar technique utilized spatially resolved 3D traits to identify 42 loci related to cardiac structure and contractility, with many loci previously implicated in cardiomyopathies.^126^

Recently, loci have also been found for previously overlooked structures. Fractal dimensions of cardiac trabeculae were found to be associated with rare variants in 56 genes involved in myocardial contractility and ventricular development and with 68 common variants.^127^^,^^128^ Increased trabeculation was most strongly associated with African ancestry, followed by physical activity, pathogenic variants for cardiomyopathy, or pre-existing disease.^128^ In the retina, GWASs of vascular fractal dimension and density identified 7 and 13 novel loci, respectively, which were enriched for pathways linked to angiogenesis and inflammation.^129^ GWASs have also identified genetic variants associated with mammographic density, with MR suggesting that a smaller mammographic density area reduces breast cancer risk.^130^

Genetics has also been used to inform the study of organ aging. Common variants in *KLF3-AS1* and *STX1* were found to be associated with brain aging, which was also highly genetically correlated with fornix volumes and the lower part of the thalamus.^131^ More recently, a study of the deviation between estimated brain age and chronological age found nine significantly associated loci and, through colocalization and MR, prioritized seven genes with genetic support as drug targets.^132^ Cardiac aging has been significantly associated with common variants at various loci (e.g., *TTN*, *ELN*, *PLCE1*, *NEURL1*, *PI15*, *SCN5A*, *CAMK2D*, and *TBX3*) and with rare variants (e.g., at *TREM2* and *MICU3*), implicating pathways related to myocardial stress response, atrial fibrosis, titin diastolic regulation, myocardial inflammation, and elastin function.^133^

A complementary approach in imaging genetics is to start with the genetic basis of diseases and investigate how these conditions first manifest in organ structure rather than beginning with the genetics of organs and working toward disease outcomes. Titin-truncating variants (TTNtv), which are linked to dilated cardiomyopathy, were found to be associated with eccentric cardiac remodeling,^134^ while *SARC-HCM-P/LP* variants, which are associated with hypertrophic cardiomyopathy, were linked to concentric remodeling, smaller right ventricular volume, higher left atrial volume, and increased trabeculation.^135^ More recent studies have shifted toward examining PGSs. For example, Pillinger et al. found that higher polygenic risk for schizophrenia was associated with adverse cardiac phenotypes, while Rabe et al. found that polygenic risk of schizophrenia was also associated with retinal thinning.^136^^,^^137^ Similarly, higher polygenic risk of Alzheimer’s disease has been associated with increased thickness of specific retinal layers.^138^

## Polygenic scores of imaging traits

GWASs have yielded many genetic variants for imaging-based organ structures, signals that have then been aggregated into PGSs that may be utilized to improve disease risk prediction or to investigate etiology using MR. A PGS for left atrial passive emptying fraction has been shown to be predictive of ischemic stroke.^139^ Similarly, PGSs of right ventricular traits have been associated with coronary artery disease and dilated cardiomyopathy,^140^ while PGSs for left ventricular traits are predictive of heart failure and dilated cardiomyopathy.^141^^,^^142^ It has been further demonstrated that the inclusion of a PGS for aortic diameter improves prediction of aortic dilation and adverse thoracic aortic events beyond clinical risk factors alone.^143^

MR has been used to investigate the causal relationships between cardiac structures and diseases. Previous studies have found that stiffer ventricles increase the risk of heart failure,^144^ higher left ventricular regional wall thickness traits increase the risk of hypertrophic cardiomyopathy,^123^ and lower left ventricle stroke volume increases the frailty index.^145^ MR has been particularly useful when applied to poorly understood structures, such as trabecular morphology, which was found to affect the risk of cardiovascular disease.^127^ In the brain, a larger intracranial volume was found to reduce the risk of developing ADHD.^146^

## Insights into multi-organ connections

The study of how different organs are correlated and intertwined has been crucially enhanced by imaging. Here, intriguingly, cardiac trabeculae have been found to share genetic loci with dendritic complexity in the brain (e.g., *MTSS1*) and with elaboration of neuronal dendritic arbors (e.g., *GOSR2)*.^127^^,^^147^ These findings are consistent with previously reported shared genetic signals between congenital heart disease (CHD) and neurodevelopmental disabilities.^148^ Phenome-wide association studies have uncovered that retinal structures have widespread links with multi-organ etiologies, including hypertension, congestive heart failure, renal failure, T2D, sleep apnea, and anemia in addition to multiple ocular conditions.^129^

When investigating the heart-brain axis, cardiac structures have been found to be genetically correlated with several neurodegenerative and psychiatric conditions and, via MR, it has been shown that adverse cardiac remodeling affects the same diseases.^149^ More recently, Zhao et al. used colocalization to find that retinal imaging traits share genetic variants with several brain disorders and complex traits and provided evidence for bidirectional genetic pathways linking retinal structure with neurological and neuropsychiatric conditions, including Alzheimer’s disease.^150^ Similarly, a study from the MULTI consortium et al. found causal relationships between imaging traits from the brain, heart and eye and diseases including Alzheimer’s disease, heart failure, and glaucoma.^151^

However, further work is needed to better understand whether these shared genetic signals arise from pleiotropy rather than from causal interactions between organs/diseases.

## Moving beyond human-defined imaging phenotypes

Although imaging genetics has shown promise in many applications, the high dimensionality and complexity of image data make determining IDPs particularly challenging. Image analysis is an essential prerequisite, typically involving steps such as filtering, registration, normalization, segmentation, and feature extraction. For feature extraction, or phenotype definition, conventional IDPs are anatomical measures defined by human experts, such as brain volume^6^ or cortical surface area and thickness.^118^ However, these are often linear measurements that reduce multidimensional, multivariate imaging data into simplified summaries, which can lead to information loss. Alternative data-driven computational techniques, such as principal-component analysis (PCA) have been applied to complex structures like the human brain.^152^ These unsupervised methods can extract comprehensive representations of high-dimensional, correlated imaging data without relying on predefined traits. The resulting IDPs can capture a substantial proportion of phenotypic variation and have proven effective in multivariate GWASs.^152^

With the rising popularity of deep learning techniques, transformer-based models have been developed to model organ structure and motion, such as MeshHeart.^153^ Latent features derived from such models show strong correlations with clinical phenotypes in phenome-wide association studies. Despite these advancements, the biological and genetic relevance of data-driven IDPs remains uncertain. Since these features are obtained through unsupervised methods, they do not necessarily reflect the genetically informative aspects of phenotypic variation. To address this, an optimized phenotyping framework^154^ was proposed and demonstrated that heritability-enriched IDPs represent genetically relevant traits and improve discovery of both common and rare genetic variants. Similar to many deep learning models, the “black box” nature of AI-derived imaging features limits direct interpretability and clinical correspondence. Techniques such as attention maps that highlight salient regions^155^ and explainable rule-based AI frameworks^156^^,^^157^ have been explored to address this limitation. However, establishing the causal validity of learned imaging features remains an open question for future research.

Recent breakthroughs in large language models or foundation models have demonstrated their potential for medical imaging analysis, including the diagnosis and prognosis of sight-threatening eye diseases^155^ and cancer imaging biomarker discovery.^158^ Furthermore, multimodal foundation models, which employ a unified architecture to learn integrated representations from multiple data types, have been developed to connect modalities such as genomics, proteomics, and transcriptomics. For example, multi-modal foundation models have linked scRNA-seq data with phenotypic information,^159^ protein sequences with biomedical texts,^160^ and single-cell multi-omics data.^161^

The impact of genetic variation on traits operates along a continuum from DNA to RNA to proteins, making it inherently complex and multi-layered. A major challenge in imaging genetics is to move beyond GWASs that focus on associations between genetic variants and imaging phenotypes and instead bridging the gap by linking cellular and molecular mechanisms to tissue- and organ-level phenotypes. At the tissue level, many molecular characteristics have been successfully inferred from cancer histology images using deep learning methods.^162^^,^^163^ Moreover, recent studies have integrated histological images with omics data across diverse organs for various tasks, including predicting spatial gene expression from histology images,^164^^,^^165^^,^^166^ tissue alignment and annotation, image-transcriptomics retrieval,^163^ disease prediction, and multicellular interaction inference.^167^ However, linking organ-level imaging phenotypes to multi-omics data remains challenging, as such phenotypes reflect integrated biological processes. Further work is needed to incorporate modalities beyond genomics, including proteomics and metabolomics.

Modeling multi-layered biological processes has motivated methodological advances. Some studies have proposed biologically knowledge-embedded models that represent hierarchical information flow among variants, genes, and multigenic systems^168^ or that integrate prior knowledge of interactions between transcription factors and pathway memberships.^169^ Other work has developed knowledge-free multi-omics variational autoencoders to identify associations between drugs and omics data through *in silico* perturbation.^170^ These methods could potentially be extended to analyze molecular mechanisms underlying imaging phenotypes. Nevertheless, coherently integrating multi-omics data with imaging phenotypes to generate new insights will require further methodological advances, potentially involving the integration of knowledge graphs and deep learning within a biologically grounded framework.

## Integrating imaging genetics, cell type enrichment, and target prioritization

Imaging genetics has demonstrated the potential to uncover physiological mechanisms and to prioritize drug targets across organs and diseases. In cardiovascular diseases, GWASs of left and right ventricular traits, combined with MR, enabled the identification of 33 plasma proteins, including drug-repurposing candidates for dilated cardiomyopathy and heart failure (e.g., IL-18R1, IL-17RA, GPC5, and LAMC2).^171^ Continuing with heart failure, CardioKG, a knowledge graph enriched with cardiac imaging traits, was able to prioritize *APP* among other genes and identify methotrexate, topiramate, and ranolazine as potential drug repurposing opportunities.^172^ In the brain, GWASs of MRI-based brain age, followed by MR and colocalization analyses, enabled the prioritization of 7 druggable targets for brain aging (e.g., MAPT, TNFSF12, GZMB, SIRPB1).^132^ Integrating cell type enrichment further refined biological mechanisms and target identification. A multi-ancestry GWAS meta-analysis of heart failure reported 47 loci, with enrichment in cardiomyocytes (using single-nucleus RNA-seq data)^13^ (Figure 1B). Subsequent proteome-wide MR prioritized 9 circulating proteins as possible therapeutic targets^13^ (Figure 1B). Beyond single-organ analysis, integrating brain, heart, and eye imaging with genetics and proteomics revealed cross-organ pleiotropic effects and enrichment of ganglia, present across all three organs, further corroborating this inter-organ axis.^151^ A downstream gene-drug-disease network analysis identified potential repurposing candidates, including AL-408, davunetide, bertilimumab, and ISIS-DMPK.^151^

Integrating data across biological scales thus enables one to address drug-target questions that would otherwise be difficult to answer. This has been made possible by past efforts to expand the multi-modality of biobanks (e.g., genetics, proteomics, and imaging) and bring single-cell technologies into both biobanks themselves as well as experimental settings (Table S1). In parallel, new statistical and computational frameworks are making it increasingly feasible to model these data jointly across modalities^173^; for example, deep convolutional neural networks that infer gene mutation status from histological images,^174^ multiplexed spatial imaging approaches such as CODEX and 3D tissue cytometry that connect single-cell phenotypes to tissue architecture,^175^^,^^176^ and emerging frameworks able to link MRI imaging with single-cell transcriptomics data.^177^ Indeed, imaging captures spatially resolved biological phenotypes, while single-cell omics provide the deep molecular resolution needed to interpret their underlying cellular and molecular bases.^178^ However, there are still key bottlenecks in integrating single-cell data and imaging genetics for therapeutic target prioritization. In particular, inferring causality across biological scales remains challenging. Limited temporal information, small numbers of participants in single-cell studies (most without linked health outcomes), and the paucity of accessible imaging data in population studies are barriers to the inference of causal effects across scales.

## Emerging directions

It seems clear that the use of human genetics to prioritize target-disease indication lists will grow in both industry and academia, potentially focusing GWAS efforts on diseases that still lack effective treatments^179^ and facilitating drug repurposing initiatives.^179^ Further, in addition to addressing health disparities arising from risk prediction, expanding ancestral diversity in enetic studies is likely to improve the identification of causal variants, especially through the inclusion of genetic data from African populations.^180^^,^^181^

In cellular genetics, focus is shifting from healthy donors to perturbations, which may better identify biological processes relevant to disease and/or drug response. Perturb-seq^182^ enables large-scale CRISPR screening coupled with single-cell transcriptomics, functionally validating GWAS-nominated genes in more realistic cellular contexts. For example, Perturb-seq, to dissect gene-regulatory networks, showed that heritability is disproportionately enriched in key transcriptional regulators in a cell type-specific manner.^183^^,^^184^ Population-scale single-cell genomics is growing rapidly with the TenK10K project,^185^^,^^186^^,^^187^ UK Biobank,^188^ and Multi-Omics Spatial Atlas Initiative (MOSIAC).^189^ Virtual cells are also pushing the boundaries of AI and biology,^190^^,^^191^ and may hold promise for optimizing the efficiency of gold standard wet-lab experiments in the future.

Novel methods, such as single-cell brain imaging transcriptomics (scBIT)^177^ or partitioned PRSs,^192^ are advancing the integration of single-cell data with organ imaging and biobank-scale data, respectively, while new atlases, such as NextBrain,^193^ are attempting to link histological reconstructions with whole-brain MRI using AI-enabled alignment and Bayesian segmentation tools. In parallel, research is rapidly increasing the integration of data from multiple organs and omics,^171^^,^^194^ particularly with respect to aging clocks.^195^ MRI-based aging clocks for different organs have recently been integrated with genetics, proteomics, and metabolomics to prioritize druggable targets for anti-aging interventions^196^ and to demonstrate the potential of drug repurposing for cross-organ diseases.^151^ Given the complexities of aging clock (a)synchronies across scales and modalities, there are likely substantial synergies for integrative analyses that leverage these population-scale cellular omics initiatives.

Taken together, the post-GWAS era has grown and matured substantially in recent years. While this review covers only a few areas that sample the different scales of molecules, cells, and organs, it is clear that genetics has a unique and crucial role to play for research at higher orders of complexity, giving us a toehold in trying to unpick disease biology and potentially downstream clinical impact.

## Acknowledgments

This work was supported by the 10.13039/501100018956NIHR Cambridge Biomedical Research Centre (NIHR203312) and 10.13039/501100023699Health Data Research UK, which is funded by the UK Medical Research Council, Engineering and Physical Sciences Research Council, Economic and Social Research Council, 10.13039/501100000276Department of Health and Social Care, Chief Scientist Office of the Scottish Government Health and Social Care Directorates, 10.13039/501100010756Health and Social Care Research and Development Division, Public Health Agency, British Heart Foundation, and Wellcome Trust. The views expressed are those of the authors and not necessarily those of the NIHR or the Department of Health and Social Care. S.I. was supported by 10.13039/501100000289Cancer Research UK (EDDPMA-May22∖100062) and by 10.13039/501100000289Cancer Research UK (EDDAPA-2024/100011). J.H.F.R. is partly supported by the 10.13039/501100000266EPSRC, the 10.13039/501100018956NIHR Cambridge Biomedical Research Centre (NIHR203312), and the British Heart Foundation Centre of Research Excellence (RE/24/130011).

## Declaration of interests

M.I. is a member of the science advisory boards of Open Targets and CIC bioGUNE, a trustee of the Public Health Genomics Foundation, and has ongoing collaborations with AstraZeneca and Nightingale Health.

## Supplemental information

Table S1. Selected datasets with imaging and genetics
